# Wise in Time

**Published:** 1871-05

**Authors:** 


### WISE IN TIME.

Peter Cooper, now in his eighty-first year, and as lithe and lively in mind, and heart, and body, as many men who are not half as old, has set the world a good example in being wise in time, instead of on the borders of eternity. He gives his money to help the poor while he is living, instead of clutching it till the last gasp of life, when he could make no further use of it.

Instead of having a great deal spent in salaries to persons to supervise his benevolences, he does that himself, leaving that much more to give. On his last birthday he gave a hundred and fifty thousand dollars, the interest of which is to purchase books of reference for mechanics from time to time, he having been a mechanic in his boyhood, and felt the great need of having books of that kind to refer to for information. This is but a part of his work, for he had already given six hundred thousand dollars to erect a building which fire cannot burn or storm destroy, where mechanics and clerks, and other poor young men and women, can, without cost, have lectures, read books, peruse newspapers and magazines, in large, well-lighted, cozy, warm rooms, day or night, summer or winter, and can, in addition, have instruction imparted in all the practical studies of life. Following him as their grand leader, come, in their turn, the Vassars, the Cornells, the Lenox, and Robert, and Peabody, and Drew, and A. T. Stewart, the prince of merchants and humanities, who freights a ship with four thousand barrels of the best flour to the suffering French, and nobody knows anything about it until the vessel has left our shores. He builds a house at the expense of millions, so that poor girls and friendless women who have to work for a living can have large, handsome, well-furnished rooms, lighted, and warmed, and cozy, and bedded, and boarded, for less than three dollars a week; a building large enough to hold hundreds and hundreds at a time. Men of money, go and do likewise in time, and thus add immeasurably to your own honor and happiness, by seeing multitudes made happy by your generosities. Remember that the Master has said it, "Inasmuch as ye have done it unto the least of these, ye have done it unto Me." Enter, therefore, immortal life.

